# Alignment strategy does not confer clinically meaningful advantages in medial pivot total knee arthroplasty: a systematic review and meta-analysis

**DOI:** 10.1186/s13018-026-06668-9

**Published:** 2026-03-24

**Authors:** Filippo Migliorini, Marco Pilone, Luise Schäfer, Raju Vaishya, Giorgio Moretti, Michael Memminger, Nicola Maffulli

**Affiliations:** 1 Department of Trauma and Reconstructive Surgery, University Hospital of Halle, Martin-Luther University Halle-Wittenberg, Ernst-Grube-Street 40, 06097 Halle (Saale), Germany; 2 Department of Orthopaedic and Trauma Surgery, Academic Hospital of Bolzano (SABES-ASDAA), Via Lorenz Böhler 5, 39100 Bolzano, Italy; 3 Department of Life Sciences, Health, and Health Professions, Link Campus University, Via del Casale Di San Pio V, 00165 Rome, Italy; 4 Department of Orthopaedic and Trauma Surgery, Eifelklinik St.Brigida, Kammerbruschstr. 8, 52152 Simmerath, Germany; 5 Residency Program in Orthopaedics and Traumatology, University of Milan, Milan, Italy; 6 Department of Orthopaedics and Joint Replacement Surgery, Indraprastha Apollo Hospital, Sarita Vihar, 110076 New Delhi, India; 7 Department of Trauma and Orthopaedic Surgery, Faculty of Medicine and Psychology, University “La Sapienza” of Rome, Via Di Grottarossa 1035, 00189 Rome, Italy; 8 School of Pharmacy and Bioengineering, Faculty of Medicine, Keele University, Stoke On Trent, ST4, 7QB UK; 9 Centre for Sports and Exercise Medicine, Barts and the London School of Medicine and Dentistry, Queen Mary University of London, Mile End Hospital, 275 Bancroft Road, London, E1 4DG UK

**Keywords:** Biomechanics, Replacement, Knee, Kinematics, Patient Reported Outcome Measures, Outcome, Mecchanic

## Abstract

**Introduction:**

Medial pivot total knee arthroplasty (TKA) has been developed to reproduce physiological tibiofemoral kinematics and improve patient satisfaction. The choice of alignment philosophy, whether mechanical alignment (MA) or kinematic alignment (KA), may influence outcomes in this context, yet evidence remains conflicting. This systematic review and meta-analysis aimed to compare clinical, functional, and radiological outcomes as well as revision rates between MA and KA in medial pivot TKA.

**Methods:**

A systematic search was conducted in PubMed, Web of Science, Embase, and Google Scholar in August 2025, following the Preferred Reporting Items for Systematic Reviews and Meta-Analyses (PRISMA) guidelines. Comparative and non-comparative clinical studies reporting outcomes of medial pivot TKA performed with either MA or KA were included. Data extracted included Knee Society Score (KSS), KSS functional subscale (KSS-F), Western Ontario and McMaster Universities Osteoarthritis Index (WOMAC), Oxford Knee Score (OKS), Forgotten Joint Score (FJS), Knee Injury and Osteoarthritis Outcome Score (KOOS), Range of Motion (ROM), and revision rates. Meta-analyses were performed when at least two studies reported comparable data.

**Results:**

Thirty-five studies comprising 5,216 patients were included, of whom 4,325 underwent medial pivot TKA with MA and 891 with KA. The dataset consisted of 12 comparative studies and 22 single-arm cohorts. Mean follow-up across studies was 62.4 months (range 12–180). At baseline, the two groups were comparable in age, sex distribution, and most outcome measures, although the KA group presented with a higher BMI and greater ROM. At final follow-up, there were no qualitatively significant differences in KSS, OKS, WOMAC, KOOS, or FJS between groups. The KA group achieved a statistically greater ROM (mean difference 4.9°, p = 0.01), and pooled analysis demonstrated a higher FJS (MD 8.36, 95% CI 4.18 to 12.55, p < 0.0001). However, the magnitude of these differences did not exceed the minimal clinically important difference. Revision rates and complication profiles were comparable between alignment strategies, although follow-up timepoints varied across studies.

**Conclusion:**

Both kinematic and mechanical alignment in medial pivot TKA yielded reliable improvements in clinical and functional outcomes, with only minor differences in motion and joint awareness that did not reach thresholds of clinical relevance. Surgical decision-making should therefore prioritise intraoperative soft tissue balance, implant-specific design, and surgeon expertise rather than the expectation of meaningful superiority of one alignment philosophy over the other.

## Introduction

Total knee arthroplasty (TKA) is a common procedure [1–3]. The development and widespread adoption of medial pivot (MP) TKA over the past two decades have marked a significant advancement in efforts to improve patient outcomes following surgery [4, 5]. MP designs aim to replicate the medial compartment’s stable pivoting motion, which is crucial for reproducing native knee biomechanics and enhancing implant performance [6, 7]. A fundamental factor influencing the success of MP TKA is the choice of surgical alignment strategy during implantation, which can significantly affect both function and implant longevity [8, 9]. Traditionally, mechanical alignment (MA) has been the gold standard, focusing on restoring a neutral mechanical axis to optimise load distribution and implant longevity [10–13]. However, this approach may not fully account for the patient’s unique anatomy, the natural joint line, and patellar tracking [14–16]. Kinematic alignment (KA) has emerged as an alternative approach, aiming to restore the native joint line and ligament balance by aligning the prosthesis according to the patient’s pre-arthritic anatomy [17, 18]. Proponents argue that KA can improve functional outcomes and enhance patient satisfaction by more accurately replicating physiological knee motion [19–24]. Nonetheless, questions remain regarding the reproducibility, long-term durability, and risk of implant loosening associated with KA, particularly in the context of medial pivot designs [25, 26]. To address some of these concerns, restricted kinematic alignment (rKA) has been proposed as a compromise, combining the benefits of KA with defined safe limits on alignment deviation to reduce the risk of implant malposition and early failure [19, 27, 28]. This hybrid technique aims to preserve more natural kinematics while maintaining mechanical principles that protect implant longevity [29, 30]. Given the contrasting philosophies behind MA, KA, and rKA, and the evolving surgical techniques, there is a need to comprehensively evaluate how these alignment strategies affect clinical outcomes, implant survival, and complication rates, specifically in MP TKAs [31–35]. Although medial pivot TKA has gained increasing popularity, the influence of alignment philosophy within this implant category remains unclear. A design-specific distinction is essential, as implant geometry fundamentally shapes kinematic behaviour and must be considered when evaluating the effect of alignment strategies. By restricting the analysis to medial pivot implants, the present study allows direct comparison of alignment strategies within a uniform kinematic framework. It avoids the confounding that arises when different implant geometries are pooled. Given the heterogeneity of previous reports, the magnitude of any potential differences remains uncertain, underscoring the need for a consolidated synthesis of the available evidence.

The purpose of this study was to determine whether kinematic alignment offers advantages over mechanical alignment in medial pivot TKA by comparing patient-reported outcome measures, range of motion, complication rates, and revision rates using data from studies that applied comparable surgical indications and outcome assessment protocols.

## Methods

### Eligibility criteria

All clinical studies investigating medial pivot TKA performed with either KA or MA were considered for inclusion. Restricted kinematic alignment (rKA) was considered a constrained variant within the kinematic alignment spectrum, consistent with previous systematic reviews. Comparative studies reporting rKA vs MA were therefore included within the kinematic alignment category when eligible. Articles published in English, German, French, Italian, or Spanish were eligible. Only studies corresponding to Levels I to III of evidence, according to the Oxford Centre for Evidence-Based Medicine [36], were included in the meta-analysis; non-comparative studies of Level IV were included in the systematic review only. Case reports, narrative reviews, editorials, expert opinions, and letters were excluded, as were studies involving animal models, in vitro experiments, cadaveric work, computational simulations, or purely biomechanical analyses. Only studies which reported the outcomes of interest at a minimum of 12 months follow-up were considered. Studies without quantitative clinical outcome data relevant to the review objectives were excluded from the final analysis.

### Search strategy

This systematic review and meta-analysis were performed in accordance with the Preferred Reporting Items for Systematic Reviews and Meta-Analyses (PRISMA) 2020 guidelines [37]. To ensure a structured approach and methodological transparency, the following framework was defined:

• Problem: end-stage knee osteoarthritis;

• Intervention: medial pivot TKA;

• Comparison: mechanical alignment vs. kinematic alignment;

• Outcomes: functional scores, range of motion, complications, and revision rates.

In August 2025, a comprehensive electronic literature search was conducted using PubMed, Web of Science, Google Scholar, and Embase. No restrictions on publication date were applied. The detailed search strings and Medical Subject Headings (MeSH) used for each database are provided in Table 1.

**Table 1:** MeSH used in the database search

Database	Search strategy (MeSH terms and keywords)
PubMed	("Arthroplasty, Replacement, Knee"[MeSH Terms] OR "total knee arthroplasty" OR "total knee replacement" OR TKA) AND (("Medial Pivot"[All Fields]) OR "medial-pivot" OR "pivot knee" OR "medial congruent") AND (("Kinematic Alignment"[All Fields]) OR KA OR ("Mechanical Alignment"[All Fields]) OR MA)
Web of Science	TS = ("total knee arthroplasty" OR "total knee replacement" OR TKA) AND TS = ("medial pivot" OR "medial-pivot" OR "pivot knee") AND TS = ("kinematic alignment" OR KA OR "mechanical alignment" OR MA)
Google Scholar	"total knee arthroplasty" AND ("medial pivot" OR "medial-pivot" OR "pivot knee") AND ("kinematic alignment" OR "mechanical alignment")
Embase	('total knee arthroplasty'/exp OR 'total knee replacement' OR TKA) AND ('medial pivot' OR 'medial-pivot' OR 'pivot knee') AND ('kinematic alignment'/exp OR 'mechanical alignment'/exp OR 'kinematic alignment' OR 'mechanical alignment')

### Selection and data collection

Two authors (F.M. and L.S.) independently screened all records identified through the database search. Titles and abstracts were examined according to the predefined eligibility criteria. Full-text articles were retrieved when the abstract could not confirm relevance. Reference lists of all included papers were manually checked to capture additional eligible studies. Any disagreements regarding study selection were resolved by discussion, with a senior author (N.M.) providing the final decision when required.

### Data items

Two reviewers (F.M. and L.S.) independently extracted the data from all included studies. All data were compiled in Microsoft Excel (version 16.0, Microsoft Corporation, Redmond, WA, USA). Baseline information included first author, year of publication, journal, level of evidence, follow-up duration, number of patients, sex distribution, and mean age. Where available, the mean BMI was also extracted. Details of the alignment strategy, implant type, outcome measures, and any complications or revisions were documented. Data concerning Knee Society Score (KSS), Western Ontario and McMaster Universities Osteoarthritis Index (WOMAC), Forgotten Joint Score (FJS), Oxford Knee Score (OKS), Knee Injury and Osteoarthritis Outcome (KOOS), range of motion (ROM), and revision rates were collected at baseline and at the final follow-up. The primary endpoint was to determine whether KA in medial pivot TKA is associated with superior clinical and functional outcomes compared to MA.

### Assessment of the risk of bias and quality of the recommendations

The risk of bias was assessed following the Cochrane Handbook for Systematic Reviews of Interventions guidelines [38]. Two reviewers (F.M. and L.S.) independently evaluated the risk of bias in the included studies. Disagreements were solved in consultation with a third senior author (N.M.). RCTs were appraised using the revised Risk of Bias assessment tool (RoB2) [39, 40] of the Cochrane tool for assessing Risk of Bias in randomised trials (RoB) [41], considering potential bias from the randomisation process, deviations from intended interventions, missing outcome data, outcome measurement, and bias in selection of the reported result. The risk of bias in non-randomised studies was evaluated using the Risk of Bias in Non-randomised Studies of Interventions (ROBINS-I) tool [42] across seven domains. These covered potential confounding factors, patient selection, classification of interventions, deviations from intended interventions, missing data, outcome measurement, and selective reporting. The ROBINS-I visualisation was produced with the Robvis software (Risk-of-Bias VISualization, Bristol, UK) [43].

### Synthesis methods

The statistical analyses were performed by the main author (F.M.) following the recommendations of the Cochrane Handbook for Systematic Reviews of Interventions [44]. For descriptive statistics, the IBM SPSS software version 25 (International Business Machines Corporation, Armonk, USA) was used. For the analysis of the baseline comparability, the mean difference (MD) effect measure and the t-test were used. Values of P < 0.05 indicate statistically significant differences between groups in the baseline demographics. For the comparisons of the outcomes of interest at the last follow-up, the MD and the odds ratio (OR) effect measures were used for continuous and dichotomic data. Standard error was also calculated. The confidence interval was set at 95% in all comparisons. Values of P < 0.05 were considered statistically significant. For the meta-analyses, the Review Manager software version 5.3 (The Nordic Cochrane Collaboration, Copenhagen) was used. The paired t-test was performed with values of P < 0.05 considered statistically significant. For continuous data, the inverse variance method with the mean difference (MD) effect measure was used. For binary data, the Mantel–Haenszel method with odd ratio (OR) effect measure was used. The confidence interval (CI) was set at 95% in all the comparisons. Heterogeneity was evaluated through Higgins-I^2^ and χ^2^ tests. If P_χ2_ > 0.05, no statistically significant heterogeneity was found. If P_χ2_ < 0.05, the heterogeneity was evaluated following the values of the Higgins-I^2^. If the Higgins-I^2^ test > 50% high heterogeneity was found. A fixed effect model was set as the default. If high heterogeneity was detected, a random model effect was used. Overall values of P < 0.05 were considered statistically significant.

## Results

### Study selection

**Fig. 1:**
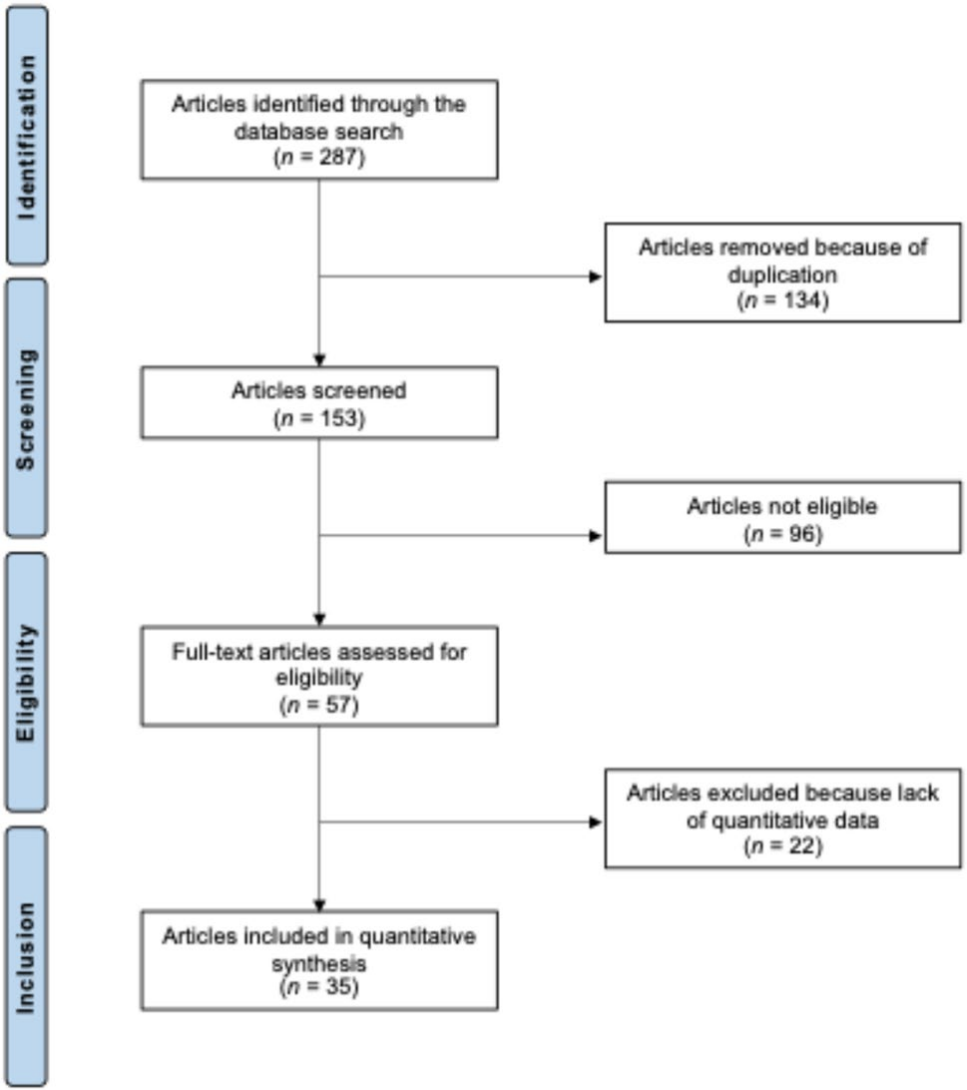
PRISMA flow chart of the literature search

The database search identified 287 records related to medial pivot TKA. After the removal of 134 duplicates, 153 records remained for screening. Titles and abstracts were reviewed, leading to the exclusion of 96 studies. The main reasons for exclusion included improper study design (N = 16), low level of evidence (N = 20), lack of application of KA or MA in medial pivot TKA (N = 43), insufficient reporting of clinical outcomes (N = 6), and language restrictions (N = 11). Twenty-two further studies were excluded after full-text review because no quantitative outcome data were available. A total of 35 studies met all inclusion criteria and were included in the analysis, comprising six RCTs, four prospective and 25 retrospective comparative and non-comparative series. The study selection process is illustrated in Fig. 1.

### Methodological quality assessment

**Fig. 2:**
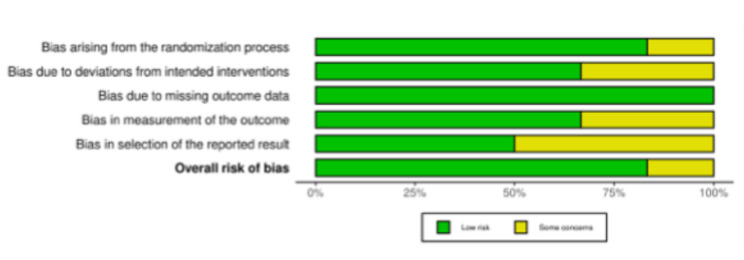
The ROB2 of RCTs

To investigate the risk of bias for RCTs included in the present systematic review and meta-analysis, the revised Risk of Bias assessment tool (RoB2) [39, 40] was performed. Across the six included randomized trials, the risk of bias was generally low. Randomization and allocation were handled appropriately in most studies, and protocols were followed without meaningful deviations. Outcome data were largely complete, and measurements were applied consistently. A few minor concerns arose, mainly around patient-reported outcomes, where blinding was not always feasible, and around selective reporting when trial registration was unclear. These limitations, however, are unlikely to have exerted a meaningful impact on the overall conclusions (Fig. 2).

**Fig. 3:**
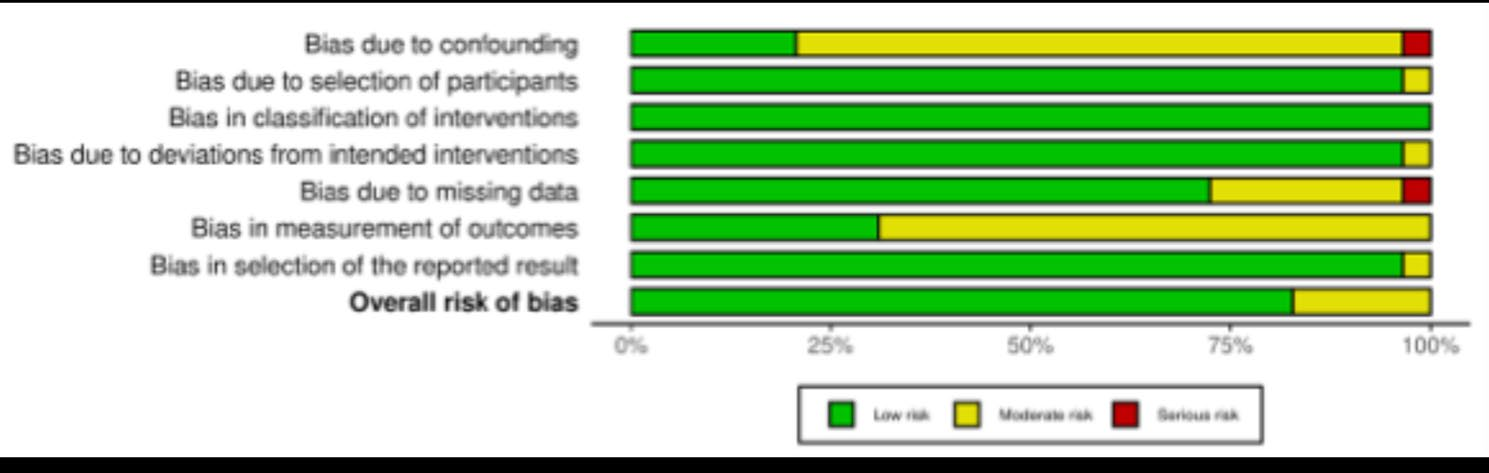
The ROBINS-I of non-RCTs

The risk of bias of non-RCTs was assessed using the ROBINS-I risk of bias tool [43]. 82.9% (29 of 35) of the included trials were non-RCTs. Most studies applied clear inclusion criteria and consistent intervention protocols, with only limited concerns related to residual confounding inherent to observational designs. In many cases, patient selection was consecutive, interventions were well described, and follow-up was complete or near complete. A few studies demonstrated particularly robust methodology, with low risk of bias in most domains. Others were rated as moderate risk, mainly given the absence of advanced methods to control for confounding, although their overall conduct and reporting were sound. Across the evidence base, measurement of outcomes, especially patient-reported scores, was commonly performed without blinding, reflecting routine clinical practice. However, standardized assessment methods and validated instruments reduce the potential impact of this limitation. Overall, the body of non-randomized evidence is methodologically solid and the overall risk of bias across the non-randomized studies was judged to be low to moderate (Fig. 3).

### Study characteristics and results of individual studies

Data from 5,216 patients were included in the present analysis. 21.3% (1,109 of 5,216) were male. A total of 4,325 patients underwent MA, while 891 received KA. The mean follow-up across the included studies was 62.4 ± 41.7 months, ranging from 12 to 180 months. The overall mean age was 70.2 ± 4.6 years, and the mean BMI was 28.4 ± 2.6 kg/m^2^. The generalities and patient characteristics are shown in Table 2.

**Table 2:** Generalities and patient characteristics of the included studies (LoE: Level of Evidence; BMI: body mass index; MA: mechanical alignment; KA: kinematic alignment)

Author	Journal	LoE	Follow-up (month)	Patients (n)	Male (n)	Age (y)	BMI (kg/m^2^)	Alignment
Bae et al., [45]	*JOA*	III	47	67	1	67.5	26.0	MA
			47	70	6	65.8	26.7	MA
Batra et al., [46]	*CiOS*	IV	60	45	5	61.7	28.4	MA
Budhiparama et al., [47]	*KSSTA*	I	48	33	9	71.0	25.8	MA
			48	33	9	71.0	25.8	MA
Chinzei et al., [48]	*The Knee*	IV	93	85	5	70.2	26.5	MA
Choi et al., [49]	*JOA*	III	60	49	6	66.7	27.6	MA
Delman et al., [50]	*KSSTA*	III	19	25	12	70.0	29.0	KA
Elorza et al., [51]	*International Orthopaedics*	III	15	25		67.0	30.8	KA
Elorza et al., [52]	*The Knee*	III	19	25	12	71.0	29.0	KA
Ettinger et al., [20]	*KSSTA*	II	48	47	38	68.8	28.1	KA
			48	51	40	63.1	29.0	MA
Fan et al., [53]	*JOA*	IV	65	59	13	65.1	-	MA
French et al., [6]	*JOA*	I	13	53	16	69.5	32.9	KA
Howel et al., [54]	*KSSTA*	III	24	117	52	68.0	30.0	KA
Hu et al., [55]	*Heliyon*	III	103	84	-	68.2	27.9	MA
			105	168	-	67.5	28.1	MA
Ishida et al., [56]	*KSSTA*	II	57	71	14	71.0	27.2	MA
Jeremìc et al., [21]	*OTSR*	III	12	24	-	70.7	30.6	KA
			12	24	-	82.5	30.0	MA
Kage et al., [57]	*J. Of experimental Orthopaedic*	III	12	19	4	74.7	25.5	MA
			12	19	5	77.2	26.2	MA
Karachalios et al., [58]	*BJJ*	IV	161	225	41	71.0	33.0	MA
Karachalios et al., [59]	*JOA*	III	180	100	31	63.2	32.0	MA
Karahan et al., [60]	*Acta Orthop Belg*	III	79	227	40	66.6	-	MA
			79	77	13	66.6	-	MA
Koutp et al., [25]	*KSSTA*	I	24	50	30	69.6	31.5	MA
			24	50	29	72.5	30.0	KA
Macheras et al., [9]	*The Knee*	III	182	176	60	73.0	28.9	MA
			182	149	50	80.0	30.1	MA
Nakamura et al., [61]	*The Knee*	IV	142	53	-	82.0	-	MA
Nakamura et al., [62]	*BMC*	III	24	45	7	74.3	25.6	MA
Niesen et al., [63]	*Acta Orthop*	III	12	35	19	67.0	31.0	KA
			12	35	21	68.0	31.0	KA
Obada et al., [64]	*International Orthopaedics*	I	24	300	-	68.4	28.5	MA
Razick et al., [65]	*JEO*	IV	30	313	131	69.0	30.0	KA
Risitano et al., [66]	*JEO*	IV	12	15	7	-	-	KA
Sosio et al., [67]	*JCM*	IV	24	55	23	71.5	29.3	KA
Ueyama et al., [68]	*Arthroplasty*	IV	142	96	6	70.2	27.2	MA
Ueyama et al., [69]	*AOTS*	III	107	153	18	77.0	23.0	MA
			60	153	23	76.0	24.0	MA
Ueyama et al., [70]	*JOA*	III	120	257	17	76.2	23.4	MA
			120	77	10	74.6	23.3	MA
Vecchini et al., [71]	*The Knee*	IV	84	160	42	71.0	-	MA
Xiang et al., [72]	*International Orthopaedics*	IV	60	1070	193	67.2	27.7	MA
Youm et al., [73]	*Knee Surgery and Related Research*	III	65	80	9	66.4	-	MA

### Baseline comparability

The two cohorts were comparable in age (p = 0.24) and male prevalence (p = 0.6). A significant difference was observed in BMI (p < 0.001), with the KA group having a higher BMI. Follow-up duration was significantly longer in the MA group (p < 0.001). At baseline, the two cohorts were comparable in KSS (p = 0.11), WOMAC (p = 0.064), OKS (p = 0.30) and FJS (p = 0.16). KA evidenced greater ROM at baseline (MD 9.5°; p = 0.004). These results are shown in Table 3.

**Table 3:** Baseline comparability (Ma: mechanical alignment; KA: kinematic alignment; BMI: body mass index; KSS: knee society score; WOMAC: western ontario and mcmaster universities osteoarthritis index; FJS: forgotten joint score; OKS: oxford knee score; ROM: range of motion)

Endpoint	MA (n = 4,325)	KA (n = 891)	*P* value
Age (*years*)	70.6 ± 5.2	69.4 ± 1.8	0.2
Sex (*male %*)	26.6 ± 38.3	33.1 ± 35.1	0.6
BMI (*kg/m*^*2*^)	27.4 ± 2.5	30.1 ± 1.3	0.0001
Follow-up (*months*)	81.7 ± 49.2	20.3 ± 10.3	0.0001
KSS	36.5 ± 11.8	43.0 ± 8.6	0.1
WOMAC	66.1 ± 9.2	53.8 ± 7.0	0.06
OKS	22.0 ± 4.7	24.1 ± 4.0	0.3
FJS	69.6 ± 10.8	75.2 ± 5.8	0.2
ROM (°)	104.2 ± 13.1	113.7 ± 5.9	0.004

### Result synthesis

The two cohorts achieved comparable outcomes in most of the evaluated endpoints. The mean postoperative KSS was 88.0 ± 7.9 in the MA group and 89.8 ± 8.3 in the KA group (MD + 1.8; p = 0.3). WOMAC was 66.1 ± 7.7 in the MA cohort and 53.8 ± 5.7 in the KA cohort, a difference that did not reach statistical significance (MD − 12.3; p = 0.06). The OKS was 42.3 ± 3.8 in MA and 42.5 ± 3.9 in KA, showing no significant difference (MD + 0.2; p = 0.9). The FJS averaged 69.6 ± 8.2 in the MA group and 75.2 ± 9.1 in the KA group, without a significant difference (MD + 5.6; p = 0.2). KOOS was 82.5 ± 6.9 in MA and 85.1 ± 7.5 in KA, also comparable between groups (MD + 2.5; p = 0.7). ROM was significantly greater in the KA cohort, with 120.5° ± 6.1 compared with 115.6° ± 7.0 in the MA group (MD + 4.9°; p = 0.01). These results are shown in greater detail in Table 4.

**Table 4:** Result synthesis (Ma: mechanical alignment; KA: kinematic alignment; KSS: knee society score; KSS-F: KSS functional subscale; WOMAC: western ontario and mcmaster universities osteoarthritis index; FJS: Forgotten joint score; OKS: oxford knee score; KOOS: knee injury and osteoarthritis outcome score; ROM: range of motion; SE: standard deviation; CI: confidence interval; MD: mean difference)

Endpoint	MA (n = 4,325)			KA (n = 891)			MD	*P* value
	Mean	SE	95% CI	Mean	SE	95% CI	MD	
KSS	88.0 ± 4.6	0.85	86.3–89.7	89.8 ± 2.9	1.43	87.0–92.6	1.8	0.3
WOMAC	66.1 ± 9.2	2.90	60.4–71.8	53.8 ± 7.0	4.04	45.9–61.7	-12.3	0.06
OKS	42.3 ± 2.8	0.80	40.7–43.8	42.5 ± 2.8	0.84	40.8–44.1	0.2	0.9
FJS	69.6 ± 10.8	3.42	62.9–76.3	75.2 ± 5.8	1.68	71.9–78.5	5.6	0.2
KOOS	82.5 ± 2.8	1.24	80.1–85.0	85.0 ± 6.0	4.25	76.7–93.4	2.5	0.7
ROM (*°*)	115.6 ± 7.1	1.28	113.1–118.1	120.5 ± 3.7	1.32	117.9–123.1	4.9	0.01

### Meta-analysis

**Fig. 4:**
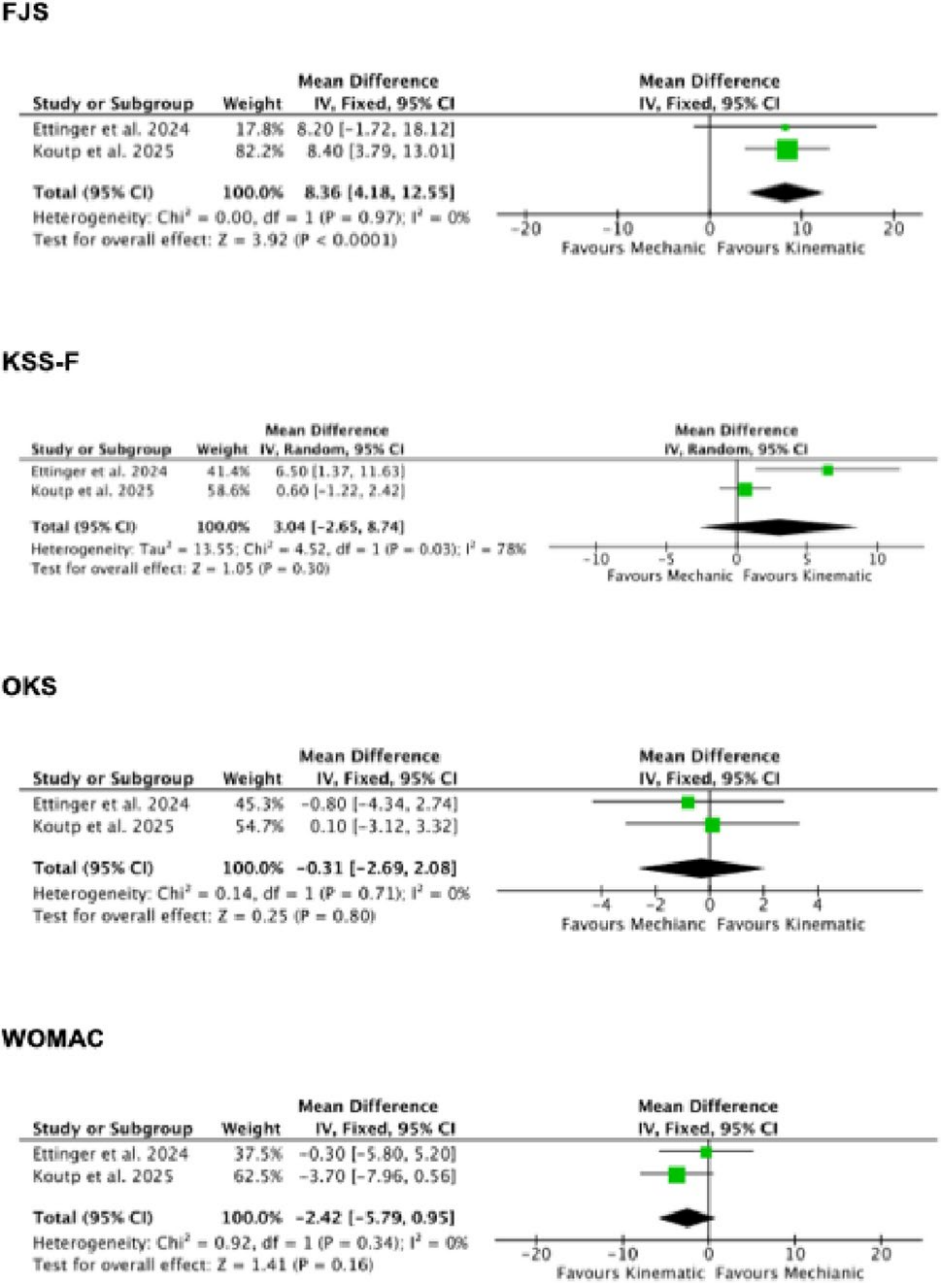
Results of the meta-analyses

Two comparative studies were included in the meta-analyses [20, 25]. The KA showed greater FJS (MD 8.36; 95% CI 4.18 to 12.55; p < 0.0001; Fig. 3). No difference was found in KSS-F, OKS, and WOMAC (Fig. 4).

## Discussion

According to the main findings of the present systematic review and meta-analysis, medial pivot total knee arthroplasty performed with KA or MA produced comparable outcomes across the main clinical endpoints. At baseline, the two groups were similar in terms of age, sex and most preoperative scores, although the kinematic cohort had a higher BMI and a significantly greater range of motion. At follow-up, both cohorts demonstrated substantial improvement in functional outcomes, with mean KSS values approaching 90 in both groups, and no significant differences were observed in OKS, WOMAC, KOOS, or FJS. The pooled analysis of randomised and non-randomised studies confirmed the absence of clinically relevant differences in most patient-reported measures. The only endpoint consistently favouring KA was the ROM, which demonstrated an average five-degree advantage compared with MA, a statistically significant difference, though its clinical importance may be debated [74]. A meta-analysis of the FJS revealed a modest statistical advantage for KA, indicating slightly greater joint awareness and satisfaction; however, the magnitude was below the thresholds generally considered clinically meaningful [75]. Revision rates and complication profiles were similar between groups, with no evidence that KA increased the risk of loosening, instability or reoperation within the available follow-up periods.

When interpreting these results, it is essential to contextualise the statistically significant differences in relation to the thresholds of minimal clinically important difference. Recent meta-analytical work on knee osteoarthritis has estimated that the MCID for knee flexion lies between 3.8° and 6.4°, values that represent the change necessary for a patient to perceive a true functional improvement in range of motion [74]. The observed difference of approximately 5° in favour of kinematic alignment therefore falls within this range, indicating that although small, the difference may indeed be clinically perceptible. However, variability in measurement techniques, patient heterogeneity and ceiling effects in modern arthroplasty must temper the interpretation. For the FJS, the MCID has been established at around 14 to 17 points, depending on the methodology [75], whereas the difference observed in the present analysis was only about 8 points. This magnitude, although statistically significant in the meta-analysis, remains below the accepted clinical threshold and should therefore not be considered clinically relevant.

The discrepancy between the systematic review and the meta-analysis regarding the FJS deserves further comment. While the systematic review synthesises data qualitatively across all available studies and showed no significant difference, the quantitative meta-analysis, which included only two comparative trials, demonstrated statistical significance. From a mathematical standpoint, the systematic review draws from a broader dataset but is limited by heterogeneity and by the inclusion of non-comparative evidence that dilutes effect estimates. The meta-analysis, on the other hand, benefits from statistical power by pooling homogeneous comparative data but is constrained by the very small number of studies included. The weight of evidence must therefore be judged by balancing breadth against precision. In this context, the systematic review reflects the global evidence base. It is less susceptible to type I error arising from small sample effects. In contrast, the meta-analysis, although methodologically robust, is more vulnerable to random variation and publication bias given the limited dataset. Consequently, greater confidence should be placed in the broader systematic review findings, while acknowledging that the meta-analytical signal may indicate a potential but as yet unconfirmed trend requiring further high-quality trials for validation. The lack of substantial differences between the two alignment philosophies when applied to medial pivot designs can be interpreted in light of biomechanical and biological principles. MA seeks to restore a neutral mechanical axis, distributing loads evenly across the tibiofemoral joint and theoretically reducing the risk of asymmetric wear, yet it has been criticised for disregarding the native joint line and the physiological obliquity of the knee [17, 76–78]. KA, in contrast, aims to replicate the patient’s prearthritic anatomy by restoring constitutional joint lines and ligament balance, which may improve kinematics and proprioception. The finding that the range of motion was slightly greater in kinematic cohorts is consistent with biomechanical studies reporting that reproducing native joint line obliquity and posterior tibial slope facilitates deeper flexion and smoother rollback of the femoral condyles [79–81]. Recent CT-based analyses by Ohyama et al. [82] further demonstrated that alignment strategy can modify posterior femoral condylar morphology even within medial pivot constructs, reinforcing that alignment influences the articular geometry that governs rollback and flexion behaviour. This effect may be amplified by the medial pivot design, which stabilises the medial compartment and allows controlled lateral rollback, mimicking native knee motion [83–85]. Zou et al. [86] likewise demonstrated that medial stability combined with controlled lateral laxity during mid-flexion correlates with favourable postoperative outcomes in medial-pivot TKA, supporting the functional relevance of this kinematic pattern. The minor advantage observed in the FJS aligns with the hypothesis that kinematic alignment enhances proprioceptive feedback by maintaining soft tissue tension closer to the native state, which may support more natural gait patterns and reduce joint awareness during activities of daily living. This interpretation is consistent with findings from Bauer et al. [87], who reported enhanced quadriceps efficiency in medial-pivot designs compared with cruciate-retaining constructs, indicating that design-specific kinematic features may exert a more substantial influence on functional performance than alignment philosophy alone. However, the absence of significant differences in most patient-reported outcomes suggests that the intrinsic stability of the medial pivot design may override minor alignment-related variations. From a biological standpoint, implant survivorship depends not only on coronal plane alignment but also on bone remodelling, cement fixation and polyethylene wear [88–93]. Contemporary evidence suggests that slight deviations from neutral alignment within the safe boundaries of restricted kinematic alignment do not compromise fixation or long-term survival, especially when combined with the medial pivot articulation, which provides intrinsic stability and balanced load transfer [4, 17, 32, 33, 94]. The comparable revision rates between kinematic and mechanical groups in this analysis support this notion. It is also relevant that most studies had relatively short to mid-term follow-up, limiting conclusions on very long-term implant survival. Furthermore, the higher BMI observed in the kinematic cohorts did not translate into worse outcomes, suggesting that medial pivot stability and balanced contact mechanics may compensate for increased loads. In summary, the available evidence indicates that both alignment philosophies, when applied to medial pivot designs, achieve reliable improvements with only minor differences in motion and joint awareness, and surgical decision making should therefore be guided by intraoperative balance, implant-specific features and surgeon expertise rather than by expectations of substantial differences in functional outcome.

Several limitations warrant consideration. The evidence base is largely composed of single-centre observational cohorts with few randomised comparisons, so selection bias, centre effects, and residual confounding by indication remain plausible despite adjustment. Although alignment methodologies were clearly described for both MA and KA, several studies did not provide systematic postoperative radiographic verification of the attained target (e.g., weight-bearing long-leg HKA and component angles, such as LDFA and MPTA, by group), which limited confidence that the intended exposure was achieved in vivo and allowed for potential misclassification. Follow-up was not balanced, with MA cohorts generally followed longer than KA cohorts, which reduces power to detect late failures under KA and may over-represent time-dependent events under MA, complicating survivorship synthesis. At the implant level, pooling first- and second-generation medial-pivot designs introduces heterogeneity in medial conformity, lateral condylar geometry, trochlear morphology, and polyethylene options; because alignment interacts with articular conformity and insert selection, such design-level effect modification can dilute or mask any true difference between KA and MA. In light of these constraints, the pooled estimates warrant cautious interpretation, and additional high-quality evidence is required before firm comparative conclusions can be drawn. Looking forward, future perspectives emphasise pre-registered, adequately powered, design-specific randomised trials; standardised radiographic reporting of achieved alignment with full distributions (HKA, LDFA, MPTA and component rotation) and balanced surveillance with long-term follow-up and time-to-event analyses.

## Conclusion

Medial pivot TKA performed with kinematic or mechanical alignment results in comparable clinical, functional, and radiological outcomes. Although the kinematic cohort achieved a statistically greater range of motion and a modest advantage in the FJS, these improvements did not exceed the minimal clinically important difference and should not be interpreted as clinically significant. Revision rates and complication profiles were similar, suggesting that both alignment philosophies are safe and effective within the medial pivot concept. The findings indicate that alignment strategy in medial pivot TKA should be selected on the basis of intraoperative ligament balance, implant characteristics, and surgeon expertise, while acknowledging that both MA and KA provide reliable outcomes without evidence of substantial clinical superiority of either of them.

## Data Availability

The datasets generated during and/or analysed during the current study are available throughout the manuscript.

## Declarations

### Use of AI

Language editing was performed using Grammarly (Superhuman Platform Inc., San Francisco, CA, USA).
